# Molar-root incisor malformation — a systematic review of case reports and case series

**DOI:** 10.1186/s12903-023-03275-6

**Published:** 2023-08-18

**Authors:** Emilija D. Jensen, Gabrielle Smart, Brianna F. Poirier, Sneha Sethi

**Affiliations:** 1 Department of Paediatric Dentistry, Women’s and Children’s Hospital, North Adelaide, South Australia Australia; 2 Adelaide Dental School, The University of Adelaide, Adelaide, South Australia Australia

**Keywords:** Children, Dental anomaly, Dental development, Odontogenesis

## Abstract

**Objectives:**

Molar-root incisor malformation (MRIM) is a seldom reported condition characterised by disturbances in root development of first permanent molars. This systematic review aimed to collate the clinical characteristics of individuals diagnosed with MRIM.

**Materials and methods:**

A systematic search strategy using PubMed, Embase, Web of Science, and SCOPUS databases was performed through to March 2023. Inclusion criteria were case reports or case series including a diagnosis consistent with MRIM. Critical appraisal for all included studies utilised the Joanna Briggs Institute (JBI) critical appraisal checklist for case reports and case series and collation of clinical characteristics was performed in JBI System for the Unified Management, Assessment and Review of Information program.

**Results:**

The search identified 157 studies from which 35 satisfied the inclusion criteria. After full-text review, a total of 23 papers described the MRIM dental anomaly and were included in this paper. A total of 130 reported cases were retrieved, with age ranging 3–32 years, and males affected 1.16:1 females. Presence of neurological conditions, premature birth history, medication, and surgery within first years of life were synthesised and described.

**Conclusions:**

The aetiology of MRIM is yet to be determined but epigenetic changes from significant medical history in the first years of life are likely to influence the development of this root malformation. First permanent molars were most commonly affected, but clinicians should be aware that permanent central incisors, primary teeth and other permanent teeth may also be affected.

**Supplementary Information:**

The online version contains supplementary material available at 10.1186/s12903-023-03275-6.

## Introduction

**Fig. 1:**
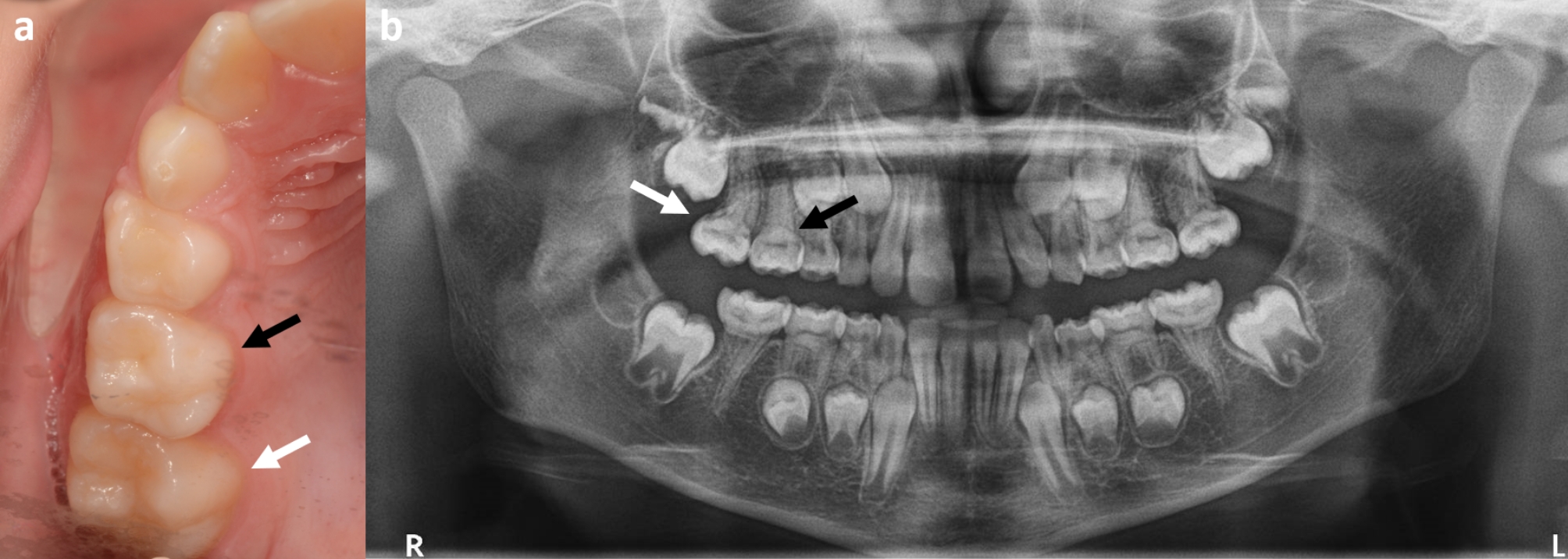
An individual in the mixed dentition with primary second molars and permanent first molars affected by molar-root incisor malformation, (**a**) a clinical photograph of the normal clinical crowns of teeth 55 (black arrow) and 16 (white arrow) and (**b**) a panoramic radiograph of the same individual with affected teeth 55 (black arrow) and 16 (white arrow) highlighting the tapered, thin roots with a constricted cemento-enamel junction consistent with molar-root incisor malformation

Molar-root incisor malformation (MRIM) (abbreviated as both MIM and MRIM) is a term used to describe a distinct tooth abnormality typically characterised by disturbances in the root development of first permanent molars (FPM). The MIM anomaly has been described as a tooth with a normal crown appearance but tapering roots with a narrowed pulp chamber (Fig. 1) [1, 2]. First permanent molars are most commonly affected but second primary molars and permanent central incisors have been reported with similar slender roots and a cervical notch. The anomaly was subsequently termed MRIM to maintain distinction from molar-incisor hypomineralisation (MIH) [3]. However, the original reference to root malformation associated with a cervical mineralised diaphragm (CMD; ectopic mineralised plates at the level of the cementoenamel junction) may be more fitting for the presentation of the majority of cases [1]. The recent increase in publication of related case reports and case series indicates that not all affected teeth are permanent or primary molars, and therefore the description may need to be modified in the future. The acronym MRIM will be used subsequently in this paper.

The clinical and radiographic appearance of MRIM has some similarities in morphology to other well-known dentine anomalies with several potential differential diagnoses (Table 1). Although dentinogenesis is thought to be relatively resistant to systemic illnesses or environmental insults, various root malformations can occur due to a result of genetic and environmental factors. Whilst dentine dysplasia (DD) type I has the most similar clinical and radiographic presentation to MRIM, it is a hereditary disorder that affects the entire dentition. Interestingly, there have been reports of DD type I with an atypical presentation and absence of a familial history — putatively misdiagnosed and potentially unreported prior to the first description of MRIM in 2014.

**Table 1:** The similarities and differences of dentine disorders that form a differential diagnosis to molar-root incisor malformation

Differential Diagnosis	Similarities to MRIM	Differences to MRIM
Dentine dysplasia type 1 OMIM #125,400	Clinically normal crown Missing or altered, thin, short root formation^†^ Pulp chamber stenosis Both primary and permanent dentitions affected	Autosomal recessive inheritance Expressed in the entire dentition
Dentine dysplasia type II OMIM #125,420	Thistle-tube deformity of pulp chambers and root canals Pulp stones Normal root length^†^	Clinical crown opalescent or translucent Autosomal dominant inheritance Unaffected permanent dentition
Dentinogenesis imperfecta type I (OMIM #125,490)	Cervical constriction with short or tapered roots Pulp chamber stenosis or obliteration[36]	Clinical crown opalescent or translucent Autosomal dominant inheritance Concurrent diagnosis of osteogenesis imperfecta
Dentinogenesis imperfecta type II (OMIM #125,490)	Cervical constriction with short or tapered roots Pulp chamber stenosis or obliteration[36]	Clinical crown opalescent or translucent Autosomal dominant inheritance
Dentinogenesis imperfecta type III (OMIM #125,500)	Primary teeth experience spontaneous pulp exposures[36]	Crowns wear rapidly Permanent teeth have large pulp chambers Amber coloured dentine A “shell” radiographic appearance due to hypotrophy of dentine Autosomal dominant inheritance
Hypophosphatemic rickets (OMIM #307,800)	Clinically normal crown Short and blunted roots[37]^†^	Enlarged pulp chambers with extension of pulp to the dentinoenamel junction and hypomineralised dentine X-linked dominant inheritance
Schimke immunoosseous dysplasia (OMIM #242,900)	Cervical constriction with short or tapered roots Obliteration or stenosis of the pulp chambers Can affect primary and/or permanent dentition[38]	Systemic conditions of spondyloepiphyseal dysplasia, renal dysfunction, T-cell immunodeficiency, and facial dysmorphism Autosomal recessive inheritance
Regional odontodysplasia	Can affect primary and/or permanent dentition Reduced thickness of dentine and irregular globular dentine Clefts between dentine tubules[35]	Typically localised to one quadrant Tooth crowns are significantly affected
Segmental odontomaxillary dysplasia	Short roots^†^ Dentine defects in coronal dentine[39]	Atypical form of fibrous dysplasia Agenesis of other teeth Enamel Defects Bone and soft tissue changes
Hypoparathyroidism and pseudohypoparathyroidism (OMIM #103,580)	Blunting of the root apices^†^ Pulp calcifications[40]	Systemic problems of end-organ resistance to parathyroid hormone Generally found to affect premolars

OMIM, Online Mendelian Inheritance in Man; MRIM, molar-root incisior malformation

^†^Root malformation can present with variable length and appearance

The aetiology of MRIM remains unclear but is considered to have an association with environmental stressors from early life. Mineralization of FPM and central incisors initiates around birth and the crowns complete mineralization around three years of age, followed by root development initiated within the fourth year of life [4]. Root development is genetically controlled by Hertwig’s epithelial root sheath (HERS) and commences development before the initiation of mineralisation of the root. The majority of MRIM cases are associated with a systemic disorder or prescription medication taken for systemic illness within the first four years of life. As MRIM is limited to specific teeth, indicative of a chronological disturbance, it appears that relevant medical history or environmental factor in the first years of life could be causative. To the authors’ knowledge, there are no systematic reviews of the literature pertaining to MRIM. Therefore, this systematic review aimed to collate the clinical characteristics of individuals diagnosed with MRIM and to identify potential patterns in teeth affected by this dental anomaly.

## Materials and methods

### Research question

This review sought to evaluate evidence addressing the following population, exposure of interest and outcome (PEO). “What are the possible factors potentially related (O) to the development of MRIM (E) in individuals (P) diagnosed with a dental anomaly consistent with MRIM?“

### Research protocol

This systematic review was registered in PROSPERO International Prospective Register of Systematic Reviews hosted by the National Institute for Health Research, registration number (CRD42021285579; March 2023) and the Joanna Briggs Systematic Reviews register. A search of these registries revealed no similar studies. This review is reported in alignment with the Preferred Reporting Items for Systematic Reviews and Meta-Analyses (PRISMA) guidelines [5] (Appendix 1).

A pre-established search strategy involving key terms and their variants was used to identify studies for inclusion in this review [6]. The search string was tailored per the design of each of the following databases: PubMed, Embase, Web of Science and Scopus (Appendix 2). For example, the search strategy used for PubMed was as follows: (“root malformation“[tiab] OR “molar incisor malformation“[tiab] OR “molar-root incisor malformation“[tiab] OR “cervical mineralised diaphragm“[tiab] OR “localised dentine dysplasia type I“[tiab] OR “non-hereditary dentine dysplasia type I“[tiab]) AND (“case report*“[tw] OR “case series“[tw]). Studies published from database inception until October 2021 were included in this review. All articles identified through the systematic search were uploaded to Endnote (Version X9, Clarivate Analytics, PA, USA).

### Literature search

Two independent reviewers (E.D.J. and S.S.) screened the titles and abstracts to assess eligibility for inclusion, with studies deemed relevant by either reviewer progressing to full-text review. The bibliographies of studies eligible for inclusion in this review were manually searched to identify additional articles. The full texts of eligible studies were uploaded to the Joanna Briggs Institute System for the Unified Management, Assessment and Review of Information (JBI-SUMARI; Joanna Briggs Institute, SA, Australia) for review. The investigator pair screened articles against the following inclusion criteria: full-text available in English; case reports or case series study design; individuals presented with a diagnosis consistent with MRIM (described as CMD, MIM, MRIM or a localised, non-hereditary DD type I).

### Data extraction

Data were extracted by two independent reviewers (E.D.J. & S.S.) into a standardised form, which included gender, age, medical history, symptoms, and teeth affected. Where data was not reported, corresponding authors were contacted by email for the opportunity to provide further information. Medical conditions were grouped by major categories. In accordance with the JBI methodology for systematic reviews of aetiology and risk, and tabulation was performed utilising the JBI SUMARI software [7]. Categorically tabulated outcomes were synthesised to provide summary findings reflective of the individual cases included in this review.

### Quality appraisal

Various tools exist for the appraisal of studies included in systematic reviews; this review utilised the JBI critical appraisal checklist for case reports and case series (Appendix 3) [7]. The tool most applicable to each paper (case report checklist unless ≥ 4 cases, then case series checklist) was utilised to maintain a high quality and relevant appraisal process. These tools include questions regarding case reporting quality, clarity, and detail. Each paper included in this review was appraised by two independent reviewers (E.D.J. & S.S.), who answered ‘yes,’ ‘no,’ ‘unclear,’ or ‘not applicable’ to the questions.

### Data analysis

Quality appraisal results were synthesised to calculate the inter-rater reliability score. The collation of medical history from case reports was manually completed by the reviewers, who pooled the findings using the JBI SUMARI software.

## Results

The search identified 157 articles, from which 82 duplicates were removed (Appendix 3). Seventy-five articles were eligible for inclusion and were title and abstract screened by two reviewers; 35 underwent full-text screening and 23 fully satisfied the inclusion criteria [1–3, 8–27]. All included studies underwent critical appraisal (Appendix 4); the inter-reviewer appraisal score was 8.39, indicating a high level of agreement between reviewers (Appendix 5). One study did not have strong methodological rigour, according to the JBI critical appraisal tool [20]. However, no articles were excluded based on a low appraisal score according to case-report or case-series criteria; 23 articles that were critically appraised were included in the review.

The review included cases from Australia, Austria, England, Norway, Slovenia, South Korea, Switzerland, and the United States (Table 2). Studies were published between 2014 and 2023 and varied in sample size (n = 1 to 38). The total number of individual cases published in the included articles was 130 (Table 3). The mean age of individuals was 8.9 ± 2.99 (range 3–23) years. Gender was reported in 122 cases (56 females) with males affected more than females at 1.16:1 ratio. Medical history was reported in the majority (95.3%) of the 130 individual cases. Neurological conditions were the most prevalent condition identified (48.5% cases). Premature birth and low birthweight occurred in 24.6% of 114 cases with birth history. Medications prescribed in the first years of life was reported in 26.0% of the 50 cases where this information was available. Surgery in the first years of life was reported in 33.8% of the 65 cases that reported sufficient history.

**Table 2:** Summary of patient cases from the literature review

Study	Country	Reported cases (n)	Male gender (n)	Neurological condition (n)	Ex-premature birth history (n)	Medication in first years of life (n)	Surgery in first years of life (n)	Age range (years)
Brusevold et al., 2017	Norway	6	2	4	2	2	2	8–12
Byun et al., 2015	South Korea	1	1	1	0	1	1	12
Choi et al., 2017	South Korea	3	1	1	1	NS^†^	1	6–9
Jensen et al. 2023	Australia	5	1	5	1	NS^†^	5	8–14
Kim et al., 2019	South Korea	38	24	15	12	NS^†^	7	3–23
Kim et al., 2020	South Korea	2	1	1	1	NS^†^	NS^†^	6–7
Korte et al. 2022	USA	2	0	2	NS^†^	2	0	7–8
Lee et al., 2014b	South Korea	12	6	10	1	NS^†^	NS^†^	4–13
Lee et al., 2015	South Korea	1	0	1	1	1	NS^†^	6
Lee et al., 2021	South Korea	1	0	NS^†^	1	1	NS^†^	8
McCreedy et al., 2016	USA	2	1	0	1	0	1	8–9
Neo et al., 2019	England	1	0	NS^†^	NS^†^	NS^†^	NS^†^	12
Park et al., 2020	South Korea	2	1	1	1	1	1	11
Pavlič et al., 2019	Slovenia	1	1	NS^†^	NS^†^	1	1	12.5
Qari et al., 2017	USA	4	2	NS^†^	NS^†^	NS^†^	NS^†^	7–15
Song et al. 2021	South Korea	2*	1	1	1	0	0	8–15
Vargo et al., 2020	USA	8	NS^†^	NS^†^	NS^†^	NS^†^	NS^†^	NS^†^
de Fátima Vieira et al., 2020	Brazil	1	1	0	NS^†^	NS^†^	NS^†^	8
Witt et al., 2014	Switzerland	2	1	0	1	2	0	8–10.5
Wright et al., 2016	South Korea	30	18	16	4	3	NS^†^	NS^†^
Youssef et al., 2019	USA	4	1	3	1	NS^†^	NS^†^	NS^†^
Yue and Kim, 2016	South Korea	1	1	NS^†^	NS^†^	NS^†^	NS^†^	13
Zschocke et al., 2017	Austria	1	1	0	0	NS^†^	1	9
	**Totals**	**130**	**65**	**61**	**29**	**14**	**20**	

^†^Not specified; *3 cases in this report were published previously and included in Wright et al., 2016 statistics

**Table 3:** Summary of key statistics from the 130 cases presented across the 23 included articles

Characteristic	Descriptive statistics
**Age** (mean ± SD, years)	*8.9 ± 2.99*
**Gender** *(n = 122)*	
*Male* *Female*	*54.1%* *45.9%*
**Medical history**	
*Neurological conditions* *Premature/low birthweight* *Medications in first years of life* *Surgeries in first years of life*	*48.5%* *24.6%* *26.0%* *33.8%*
**Affected teeth**	
*One or more first permanent molars (FPM)* *All four FPM* *Mandibular FPM only* *Maxillary FPM only* *Upper permanent central incisors* *All second primary molars*	*99.2%* *39.2%* *0.02%* *0%* *16.9%* *39.1%*

In addition to reported medical history, many papers discussed potential aetiology such as brain/neurological conditions,[15, 26, 28–31]. Other suggestions were the exposure to drugs in early infantile life either orally, intravenously or through breastfeeding, including antibiotics and corticosteroids [9, 15, 20, 25, 29, 32]. Some studies discussed the impact of specific conditions such as PHACE syndrome [22] and ciliopathy with homozygous mutation in *TCTEX1D2* [24], while other studies specified that that not all included patients had complex medical histories [33].

Aetiology proposals included epigenetic considerations such as a suggestion that early disturbances to epithelial-mesenchymal cross talk may affect differentiation of odontoblasts [28], phenotypic differences in monozygous twins with only one twin expressing MIRM [33], damage to the base of the dental papilla vascular plexus during crown development [12, 22, 32], epigenetic changes related to neurogical conditions or significant medical condition during tooth development [29, 34] or a suggestion that a spectrum of dentine disorders exist and that MRIM could be considered as a localised, non-hereditary variant [19]. Specific genes were postulated due to similarities to other forms of short or absent root in murine models with defects including Nfic gene,14 the Ptc gene, and the Dkk1 gene [32], or genes involving the molecular control of root form with Hedgehog and FGF suggested relating to the regulation of Hertwig’s epithelial root sheath [31] and one study completed genetic DNA extraction from blood samplex with exon sequencing which found a homozygous deletion of exon 3 and adjacent intronic sequences in introns 2 and 3 of TCTEX1D2, associated with the dysfunction of primary cilia and Jeune syndrome but proposed as a potential related mechanism for MRIM [24].

Radiographic investigations were mostly plain film panoramic radiographs. In addition to this, some studies provided CBCT evaluation [9, 12, 15, 21, 23, 32, 33] and some used microCT investigations [12, 25, 32–34]. MicroCT findings included numerous root canals not connected to the pulp chamber [28], root canals that failed to differentiate into mature canals [33], porous channels between the CEJ and the inner pulp [25], accessory canals from the lower and middle layers of the pulpal floor [34], and ectopic mineralised plates at the level of the CEJ with dense networks of delicate soft tissue canals [32].

Histopathologic investigations were typically using light microscopy [18, 19, 24, 25, 28, 31, 32] but scanning electron microscopy (SEM) and transmission electron microscopy (TEM) were also used [18, 32]. These methods found irregular and globular appearance to dentine [28, 32], amorphous dentine [25, 32, 34], dentine with channels between the outer coronal dentine and inner furcation [25] and folded dentine with unusual tissue filling the majority of the pulp chamber [18]; pulp stones [19], dysplastic pulp features [31] and abnormal morphology at the CEJ were also reported [24]. Other investigations included immunohistochemical staining with findings of moderately expressed mean active menerlisation of dentine in the upper pulpal floor [34] as well as polymerised chain reaction (PCR) and 16s rRNA sequencing of the microbiome expressing pathogens consistent with localised juvenile periodontitis [14].

Clinical consequences included severe pain, abscesses, draining sinus, pus and swelling [9, 14, 17, 18, 23, 25, 27, 28, 33]. Periapical cyst, endodontic and periodontic complications, mobility and early loss of teeth were also reported [11, 24, 29]. Management by extraction was frequently the outcome of MRIM [12, 14, 25, 27–29, 34], but endodontic therapy was also provided in some cases [9, 23]. Active surveillance was recommended in some studies [15].

**Fig. 2:**
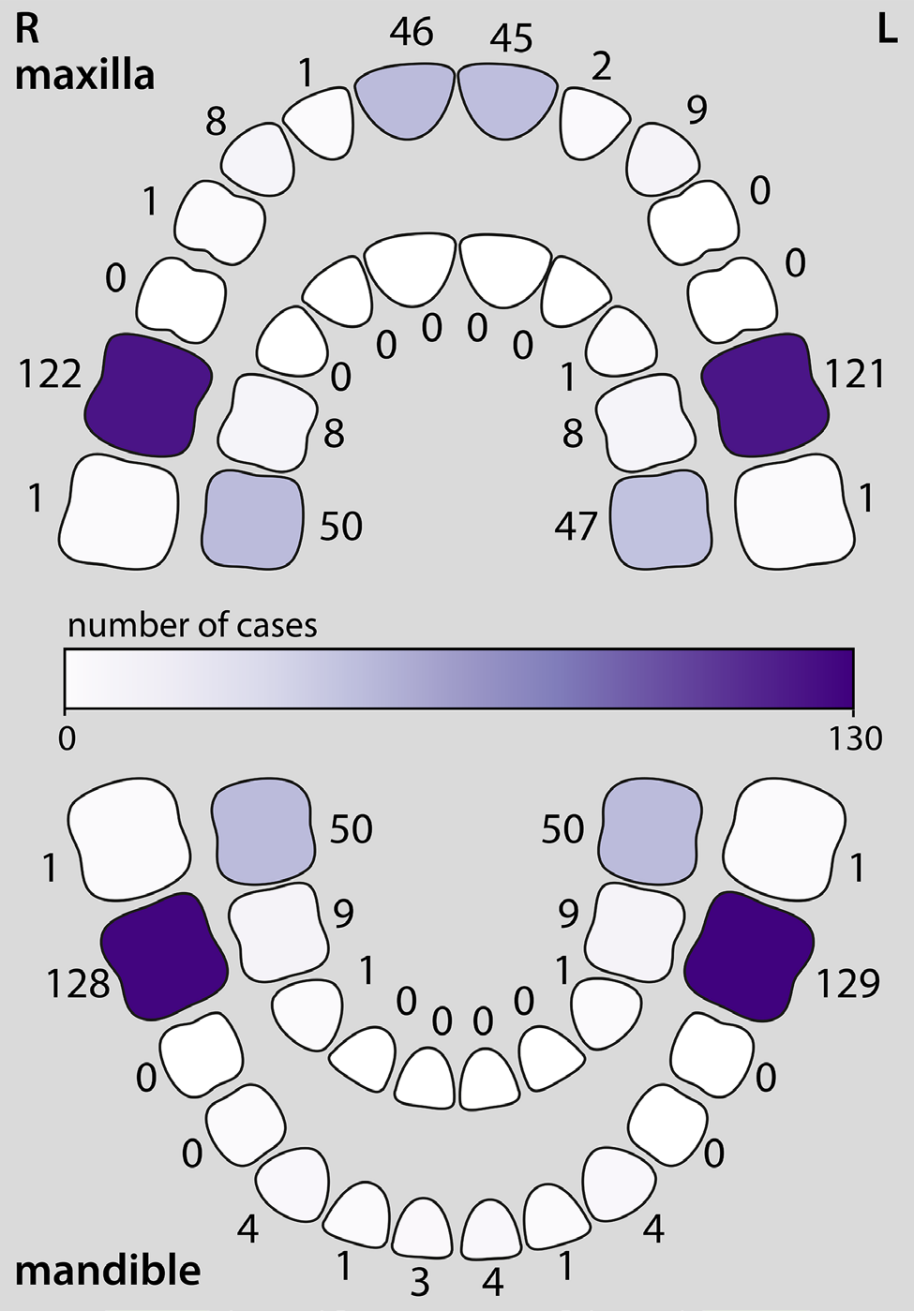
Pictorial representation of permanent (outer arch) and primary (inner arch) teeth affected by molar-root incisor malformation. The number of reported cases (out of a total of 130 cases) is noted and shaded as a gradient corresponding to the scale in the centre of the diagram

Affected teeth reported for all cases is summarised in Fig. 2. At least one FPM was affected in all but one case, but FPM only were reported in 51 cases (39.2%)[19]; of these, three cases had only mandibular FPM affected while no cases had exclusively maxillary FPM affected. Twenty-two (16.9%) cases had affected FPM and upper permanent central incisors only. Forty-four (33.8%) cases had affected FPM and primary molars. All primary second molars and FPM were affected in 51 (39.2%) cases, and 51 (39.2%) cases had an affected FPM in addition to at least one primary tooth.

## Discussion

The results of this systematic review of available case reports and case series provide evidence that although teeth affected by MRIM are most commonly FPM, various other permanent teeth including central incisors, and second molars and also primary teeth, may be affected. The reported MRIM anomalies can be viewed as a chronological continuum with the unifying features being the phenotype of cervical constriction and malformation of the root. The majority of cases in the published literature have a significant medical history event within the first four years of life when root initiation and formation of the affected teeth typically occurs. The radiographic features of MRIM are indistinguishable from DD type I and it has previously been postulated to be a non-hereditary, epigenetic cause to the same entity [19].

The clinical and radiographic presentations of MRIM have underlying consistency [10]. The crowns of FPM and second primary molars affected with MRIM are visually normal in appearance with regard to colour and shape. Teeth with MRIM typically have constriction at the CEJ (visually and radiographically) and roots that are thin, tapered, bent, and often one or more roots (of a multi-rooted tooth) has a short, atypical root form. In molars, a long distal root and irregular mesial roots have been described, as well as a soft tissue nodule between the short mesial roots [8]. Other clinical findings include increased caries experience, poor oral hygiene around affected teeth, spontaneous pain, mobility, and periodontitis with exposed furca [2]. Incisors and canines may present with a wedge-shaped defect located one-third to one-half in the cervical portion of the tooth crown [11, 35]. Conversely, the crowns of MRIM-affected FPM appear to have a normal shape (Fig. 2a). Radiographically, there is clear definition of coronal enamel and dentine (Fig. 2b), however, the affected roots may be difficult to visualise on the film with tapered, divergent, thin or shortened root systems [2]. A marked reduction in the radiographic height of the pulp cavity leads to an appearance of a narrow slit or constriction into a linear form [1, 36]. The initial description of MRIM included ectopic, mineralised plates at the level of the cemento-enamel junction (CEJ) with a radiodensity between that of enamel and dentine, the CMD [1]. The CMD areas contain dense networks of hard tissue but also contain soft tissue canals and have been described as being porous [1, 13]. It was postulated that the CMD forms in response to damage to the vascular plexus at the dental papilla base. Pulp stones within the pulp cavity of teeth with MRIM have also been reported [12].

All studies included were case reports and case series in design and we were therefore unable to provide a comprehensive review of aetiology, prevalence, and clinical presentation. Information regarding the teeth affected by MRIM was presented in all included articles, providing an understanding of the distribution of teeth affected by MRIM thus far. However, there were inconsistencies between diagnostic tests and imaging to provide substantive clinical information. Some studies used panoramic radiographs, cone-beam computed tomography, micro computed tomography, scanning electron microscopy, ground sections and immunohistochemical studies, or a combination of techniques, to provide detailed information on the affected specimens. However, many of these techniques rely on extraction of the affected teeth. These studies were recognised as contributing to the understanding of MRIM, but not enough studies included these techniques to allow for meta-analysis of the results and meaningful characterisation of MRIM by technique type.

High heterogeneity of case reports and case series is a limitation of this systematic review. Many different populations of varying sample sizes were represented among the included case reports and case series. Inconsistencies with who diagnosed the condition as well as how the condition was defined and diagnosed also may have contributed to the heterogeneity. When the article included four cases or fewer, it was considered as a case report for critical appraisal purposes as the population and type of sampling was not usually described. There was variability in the included details of the individuals’ medical history which was retrospectively obtained. Detailed medical history within the first years of life may help to understand the aetiology underlying MRIM. The most reported findings in this review included presence of neurological conditions, premature birth and low birthweight, prescription medication, and surgery in the first years of life. Future research should specifically include the presence or absence of these conditions among other potential contributory factors to ascertain the cause(s) of this dental anomaly.

The findings of this systematic review of case reports and case series add to the growing body of evidence that supports an association between MRIM and significant medical event in the first four years of life. The most significantly finding of this review was the distribution of teeth affected in the primary and permanent dentitions. Our search captured 130 cases of MRIM in the literature, but further comprehensive studies are warranted to provide high-quality evidence for techniques in clinical description of MRIM, aetiology, and prevalence.

Molar-incisor root malformation is a dental anomaly predominately affecting FPM but also affecting permanent central incisors, primary teeth, and other permanent teeth to a lesser extent. Although the aetiology of MRIM is uncertain, epigenetic changes from significant medical events, including neurological conditions, prematurity and low birth weight, surgery, and prescription of medication in the first years of life, is likely to influence the development of MRIM. Clinicians should be aware of MRIM as a separate dental anomaly to inherited conditions such as DD and DGI with no apparent hereditary cause.

### Electronic supplementary material

Below is the link to the electronic supplementary material.

Supplementary Material 1: PRISMA Statement

Supplementary Material 2: Logic grid and search terms

Supplementary Material 3: PRISMA 2020 flow diagram for this review

Supplementary Material 4: Agreed responses between two reviewers for critical appraisal for case reports and case series

Supplementary Material 5: Inter-reviewer reliability for critical appraisal

## Data Availability

The datasets used and/or analysed during the current study available from the corresponding author on reasonable request.
