# Correction: Molar-root incisor malformation — a systematic review of case reports and case series

**DOI:** 10.1186/s12903-023-03593-9

**Published:** 2023-11-24

**Authors:** Emilija D. Jensen, Gabrielle Smart, Brianna F. Poirier, Sneha Sethi

**Affiliations:** 1 Department of Paediatric Dentistry, Women’s and Children’s Hospital, North Adelaide, South Australia Australia; 2 Adelaide Dental School, The University of Adelaide, Adelaide, South Australia Australia

**Correction: BMC Oral Health (2023) 23:576**

**https://doi.org/10.1186/s12903-023-03275-6**

In the original publication of the article [1], the author has noticed the below two errors after publication.

- The figure legends for Figs. 1 and 2 are the wrong way round.
- The ‘number of cases’ in Fig. 2 in the published manuscript is incorrect (*n* = 121), whereas the newer version is correct and corresponds with the content of the article (*n* = 130).

**Fig. 1:**
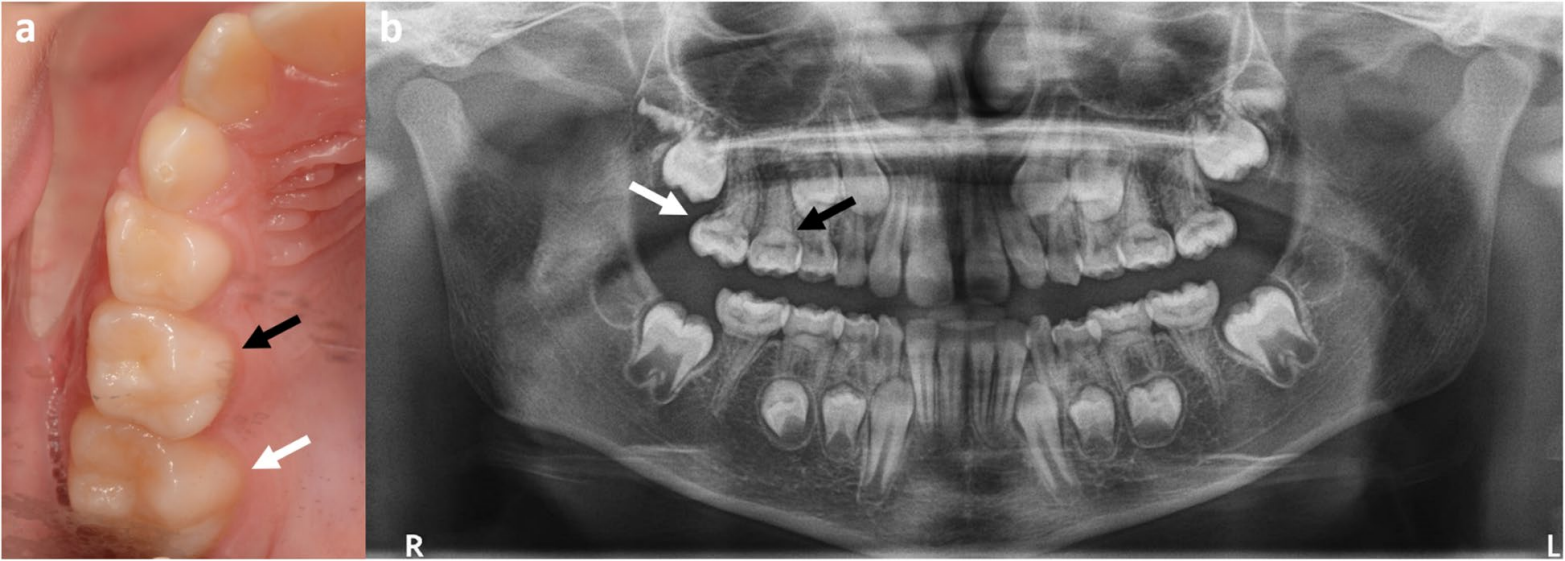
An individual in the mixed dentition with primary second molars and permanent first molars affected by molar-root incisor malformation, **a** a clinical photograph of the normal clinical crowns of teeth 55 (black arrow) and 16 (white arrow) and **b** a panoramic radiograph of the same individual with affected teeth 55 (black arrow) and 16 (white arrow) highlighting the tapered, thin roots with a constricted cemento-enamel junction consistent with molar-root incisor malformation

**Fig. 2:**
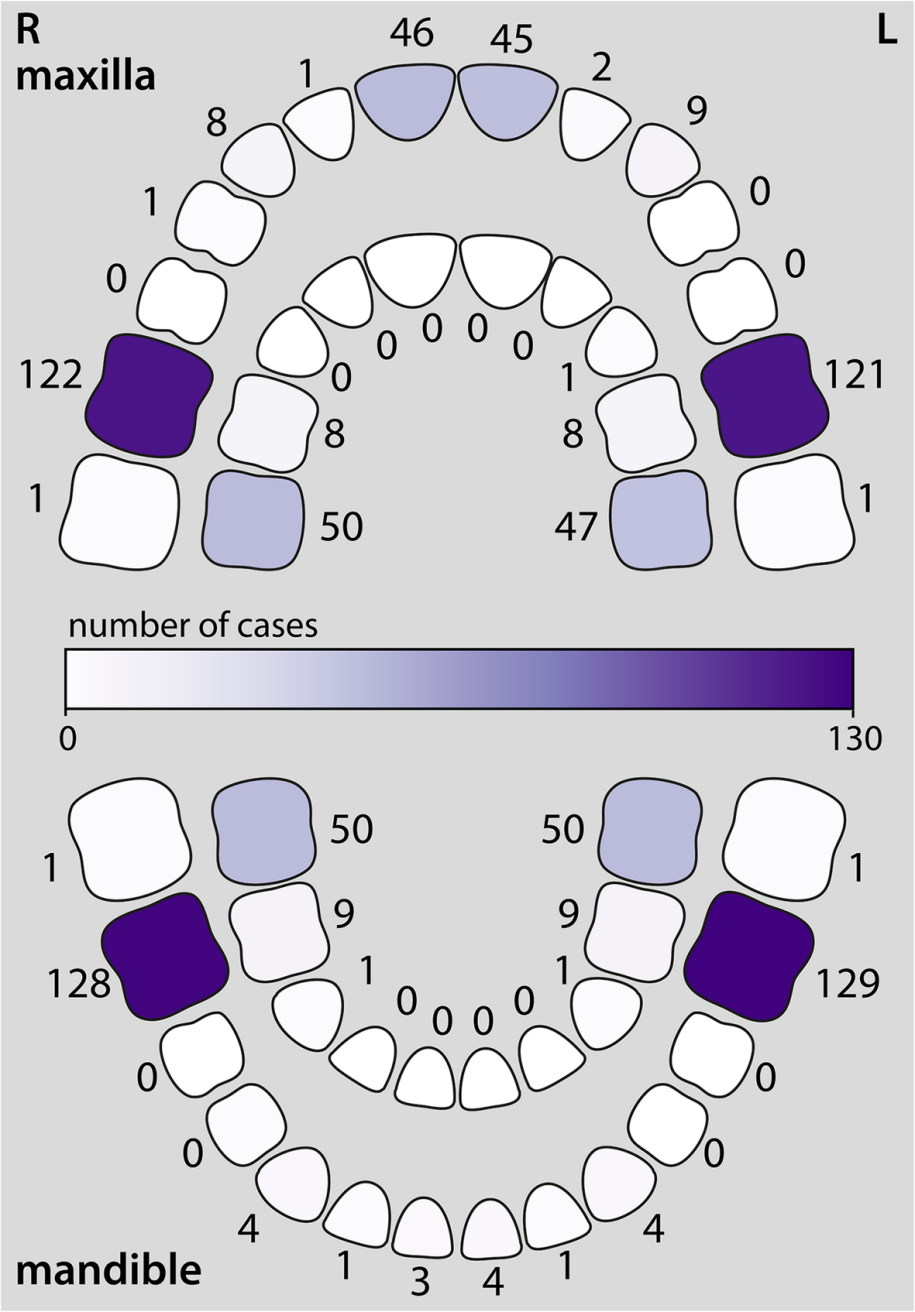
Pictorial representation of permanent (outer arch) and primary (inner arch) teeth affected by molar-root incisor malformation. The number of reported cases (out of a total of 130 cases) is noted and shaded as a gradient corresponding to the scale in the centre of the diagram

The corrected Figs. 1 and 2 are given below.

The original article has been corrected.
