# Supplementary material for: Molar-root incisor malformation — a systematic review of case reports and case series
Source: BMC Oral Health. 2023 Aug 18;23:576. doi: 10.1186/s12903-023-03275-6 (PMC10439578; doi:10.1186/s12903-023-03275-6)
Supplement: Supplementary file 2 — Supplementary Material 2: Logic grid and search terms [file 12903_2023_3275_MOESM2_ESM.docx]

**Supplemental File 2: Logic grid and search terms (Search 6 March 2023)**

PubMed – 23

| Root malformation | Case report/series |
| --- | --- |
| "root malformation"[tiab] OR "molar incisor malformation"[tiab] OR "molar-root incisor malformation"[tiab] OR "cervical mineralised diaphragm"[tiab] OR "localised dentine dysplasia type I"[tiab] OR "non-hereditary dentine dysplasia type I"[tiab] | "case report*"[tw] OR "case series"[tw] |

Embase – 32

| Root malformation | Case report/series |
| --- | --- |
| "root malformation”:tiab OR "molar incisor malformation":tiab OR "molar-root incisor malformation":tiab OR "cervical mineralised diaphragm":tiab OR "localised dentine dysplasia type I":tiab OR "non-hereditary dentine dysplasia type I":tiab | "case report*"[tw] OR "case series"[tw] |

Scopus – 43

| Root malformation | Case report/series |
| --- | --- |
| "root malformation” OR "molar incisor malformation" OR "molar-root incisor malformation" OR "cervical mineralised diaphragm" OR "localised dentine dysplasia type I" OR "non-hereditary dentine dysplasia type I" | "case report*" OR "case series” |

Web of science – 49

| Root malformation | Case report/series |
| --- | --- |
| "root malformation” OR "molar incisor malformation" OR "molar-root incisor malformation" OR "cervical mineralised diaphragm" OR "localised dentine dysplasia type I" OR "non-hereditary dentine dysplasia type I" | "case report*" OR "case series” |
