# Supplementary material for: Molar-root incisor malformation — a systematic review of case reports and case series
Source: BMC Oral Health. 2023 Aug 18;23:576. doi: 10.1186/s12903-023-03275-6 (PMC10439578; doi:10.1186/s12903-023-03275-6)
Supplement: Supplementary file 3 — Supplementary Material 3: PRISMA 2020 flow diagram for this review [file 12903_2023_3275_MOESM3_ESM.docx]

**Appendix 3: PRISMA 2020 flow diagram for this review**

**Identification of studies via other methods**

**Identification of studies via databases and registers**

Records identified from:

Citation searching (n = 3)

Records removed *before screening*:

Duplicate records removed (n = 82)

Records identified from:

Databases (n = 157)

*PubMed (n = 25)*

*Scopus (n = 47)*

*Web of Science (n = 53)*

*Embase (n = 32)*

**Identification**

Records screened:

(n = 75)

Records excluded:

(n = 33)

Reports not retrieved:

(n = 0)

Reports sought for retrieval:

(n = 3)

Reports sought for retrieval:

(n = 42)

Reports not retrieved:

(n = 7)

**Screening**

Reports excluded:

(n = 0)

Reports assessed for eligibility:

(n = 3)

Reports assessed for eligibility:

(n = 35)

Reports excluded: (n = 9)

Ineligible study design (n = 0)

Ineligible phenomena of interest (n = 8)

Ineligible condition (n = 1)

Studies included in review:

(n = 23)

**Included**

*From:*  Page MJ, McKenzie JE, Bossuyt PM, Boutron I, Hoffmann TC, Mulrow CD, et al. The PRISMA 2020 statement: an updated guideline for reporting systematic reviews. BMJ 2021;372:n71. doi: 10.1136/bmj.n71. For more information, visit: <http://www.prisma-statement.org/>
