# Supplementary material for: Molar-root incisor malformation — a systematic review of case reports and case series
Source: BMC Oral Health. 2023 Aug 18;23:576. doi: 10.1186/s12903-023-03275-6 (PMC10439578; doi:10.1186/s12903-023-03275-6)
Supplement: Supplementary file 4 — Supplementary Material 4: Agreed responses between two reviewers for critical appraisal for case reports and case series [file 12903_2023_3275_MOESM4_ESM.docx]

**Appendix 4: Agreed responses between two reviewers for critical appraisal for case reports and case series**

#### Case Reports

| **Study** | **Q1** | **Q2** | **Q3** | **Q4** | **Q5** | **Q6** | **Q7** | **Q8** |
| --- | --- | --- | --- | --- | --- | --- | --- | --- |
| *Byun et al. 2015* | Y | Y | Y | Y | Y | Y | N/A | Y |
| *Kim et al. 2020* | Y | Y | Y | Y | Y | Y | N/A | Y |
| *Korte et al. 2022* | Y | Y | Y | Y | Y | Y | Y | Y |
| *Lee et al. 2021* | Y | U | U | Y | Y | Y | N/A | Y |
| *Lee et al. 2015* | Y | Y | Y | Y | Y | N/A | N/A | Y |
| *Lee et al. 2014* | Y | Y | Y | Y | Y | Y | N/A | Y |
| *McCreedy et al. 2016* | Y | Y | Y | Y | Y | Y | N/A | Y |
| *Neo et al. 2019* | Y | U | Y | Y | Y | Y | N/A | Y |
| *Park et al. 2020* | Y | Y | U | U | Y | Y | Y | Y |
| *Pavlič et al. 2019* | Y | Y | Y | Y | Y | Y | N/A | Y |
| *Qari et al. 2017* | Y | N | Y | Y | Y | Y | N/A | Y |
| *Witt et al. 2014* | Y | Y | Y | Y | Y | Y | N/A | Y |
| *Youssef et al. 2019* | Y | Y | Y | Y | Y | Y | N/A | Y |
| *Yue and Kim 2016* | Y | Y | Y | U | Y | Y | N/A | Y |
| *Zschocke et al. 2017* | Y | Y | Y | Y | Y | Y | N/A | Y |
| *Choi et al. 2017* | Y | Y | Y | Y | Y | Y | N/A | Y |
| *Vieira 2020* | Y | U | Y | Y | Y | Y | N/A | Y |
| % | 100.0 | 76.5 | 88.2 | 88.2 | 100.0 | 94.1 | 11.8 | 100.0 |

#### Case Series

| **Study** | **Q1** | **Q2** | **Q3** | **Q4** | **Q5** | **Q6** | **Q7** | **Q8** | **Q9** | **Q10** |
| --- | --- | --- | --- | --- | --- | --- | --- | --- | --- | --- |
| *Brusevold et al. 2017* | U | Y | Y | N | Y | Y | Y | Y | Y | N/A |
| *Jensen et al. 2023* | Y | Y | Y | Y | Y | Y | Y | Y | Y | Y |
| *Kim et al. 2019* | Y | Y | Y | Y | Y | Y | Y | Y | Y | Y |
| *Song et al. 2021* | Y | U | Y | N | N | Y | Y | Y | Y | N/A |
| *Vargo et al. 2020* | Y | U | Y | Y | Y | N | N | N | Y | Y |
| *Wright et al. 2016* | Y | U | N | Y | Y | Y | Y | Y | Y | Y |
| % | 83.3 | 50.0 | 83.3 | 66.7 | 100.0 | 83.3 | 83.3 | 83.3 | 100.0 | 66.7 |
