# Supplementary material for: Molar-root incisor malformation — a systematic review of case reports and case series
Source: BMC Oral Health. 2023 Aug 18;23:576. doi: 10.1186/s12903-023-03275-6 (PMC10439578; doi:10.1186/s12903-023-03275-6)
Supplement: Supplementary file 5 — Supplementary Material 5: Inter-reviewer reliability for critical appraisal [file 12903_2023_3275_MOESM5_ESM.docx]

# **Appendix 5: Inter-reviewer reliability for critical appraisal**

| **Study** | **Number of questions in agreement** | **Number of questions in disagreement** | **Score** |
| --- | --- | --- | --- |
| *Brusevold, I. J, 2017* | 8 | 2 | 8 |
| *Byun C, 2015* | 9 | 1 | 9 |
| *Choi S, 2017* | 9 | 1 | 9 |
| *Jensen et al. 2023* | 9 | 1 | 9 |
| *Kim J, 2019* | 9 | 1 | 9 |
| *Kim MJ, 2020* | 9 | 1 | 9 |
| *Korte et al. 2022* | 10 | 0 | 10 |
| *Lee H, 2021* | 8 | 2 | 8 |
| *Lee H, 2015* | 8 | 2 | 8 |
| *Lee H, 2014* | 10 | 0 | 10 |
| *McCreedy C, 2016* | 9 | 1 | 9 |
| *Neo HL, 2019* | 8 | 2 | 9 |
| *Park S, 2020* | 7 | 3 | 7 |
| *Pavlic A, 2019* | 9 | 1 | 9 |
| *Qari, H, 2017* | 5 | 5 | 5 |
| *Song et al. 2021* | 9 | 1 | 9 |
| *Vargo RJ, 2020* | 6 | 4 | 6 |
| *Veira FGF, 2020* | 8 | 2 | 8 |
| *Witt CV. 2014* | 9 | 1 | 9 |
| *Wright JT, 2016* | 7 | 3 | 7 |
| *Youessef MJ, 2019* | 8 | 2 | 8 |
| *Yue W, 2016* | 9 | 1 | 9 |
| *Zschocke J, 2017* | 9 | 1 | 9 |
| Mean |  |  | **8.39** |
